# Mid-Term Clinical Outcomes of Pullout Repair Combined with Osteochondral Autograft Transplantation for Medial Meniscus Posterior Root Tears with Focal Cartilage Defects: A Treatment-Stratified Cohort Study

**DOI:** 10.3390/bioengineering13030343

**Published:** 2026-03-16

**Authors:** Naoki Akura, Koki Kawada, Yuki Okazaki, Keisuke Kintaka, Yuya Kodama, Toshiki Kohara, Takayuki Furumatsu

**Affiliations:** 1 Department of Orthopaedic Surgery, Graduate School of Medicine, Dentistry, and Pharmaceutical Sciences, Okayama University, 2-5-1 Shikata-cho, Kita-ku, Okayama City 700-8558, Okayama, Japan; pevz5ffn@s.okayama-u.ac.jp (N.A.); te4sf.stra.16@gmail.com (K.K.); kohatoshi10@gmail.com (T.K.); takamatino@gmail.com (T.F.); 2 Department of Orthopaedic Surgery, Tsuyama Chuo Hospital, 1756 Kawasaki, Tsuyama City 708-0841, Okayama, Japan; 3 Department of Orthopaedic Surgery, Kochi Health Sciences Center, 2125-1 Ike, Kochi City 781-8555, Kochi, Japan; me20028@s.okayama-u.ac.jp; 4 Department of Orthopaedic Surgery, Mizushima Central Hospital, 4-5 Mizushima Aobacho, Kurashiki City 712-8064, Okayama, Japan; ykodama314@gmail.com; 5 Department of Orthopaedic Surgery, Japanese Red Cross Okayama Hospital, 2-1-1 Aoe, Kita-ku, Okayama City 700-8607, Okayama, Japan

**Keywords:** cartilage lesion, joint preservation, medial meniscus posterior root tear, osteochondral autograft transplantation, pullout repair, unicompartmental knee arthroplasty

## Abstract

Medial meniscus posterior root tears (MMPRTs) with focal cartilage defects present a therapeutic challenge, even in neutral-to-mild varus knees. Although transtibial pullout repair is standard for MMPRTs without advanced osteoarthritis, coexisting cartilage lesions may compromise outcomes and prompt unicompartmental knee arthroplasty (UKA). Combining pullout repair with osteochondral autograft transplantation (OAT) may offer a joint-preserving alternative by restoring meniscal hoop stress and reconstructing focal osteochondral defects. However, supporting evidence is limited. We retrospectively analyzed 150 patients treated surgically for MMPRT between 2015 and 2019, divided into three groups: pullout repair with OAT (Group O, *n* = 6), pullout repair alone (Group P, *n* = 120), and UKA (Group U, *n* = 24), with OAT being applied only in carefully selected patients based on strict clinical and radiographic indications. Clinical outcomes were assessed preoperatively, at 1 year, and at final follow-up (mean, 4.2–5.8 years). The primary outcome was the final clinical score, and secondary outcomes were changes from baseline. All groups improved postoperatively. Group O showed marked improvement in Knee Injury and Osteoarthritis Outcome Score—Symptom and Visual Analogue Scale—Pain score, achieving outcomes comparable to Group U at final follow-up. Group P showed consistent improvement from baseline. Radiographically, mild osteoarthritis progression was observed in Group O. Given the small sample size in Group O and the retrospective design, the findings are exploratory and warrant confirmation in larger prospective studies.

## 1. Introduction

Medial meniscus (MM) posterior root tears (PRTs) disrupt the hoop stress-converting function of the meniscus, leading to increased tibiofemoral contact pressure and accelerated progression of knee osteoarthritis (OA) [1,2]. Loss of posterior root integrity compromises circumferential hoop tension, resulting in meniscal extrusion and diminished load distribution. This mechanical failure increases focal cartilage stress and adversely affects the biological environment required for cartilage homeostasis and repair [1]. Furthermore, altered joint contact mechanics have been demonstrated even at low flexion angles during simulated gait, indicating that root insufficiency leads to abnormal loading patterns under physiological conditions [2]. Conservative treatment of MMPRTs has been associated with poor clinical and radiographic outcomes [3]. Consequently, surgical repair—particularly anatomic transtibial pullout repair—has become the preferred treatment for symptomatic MMPRTs without advanced OA, as supported by recent consensus statements and mid- to long-term outcome studies [4,5,6,7].

Despite advances in repair techniques, OA progression after MMPRT repair is not completely prevented, partly due to persistent MM extrusion and pre-existing cartilage damage [8,9]. Although anatomic root repair aims to restore hoop tension and normalize tibiofemoral load transmission [1], incomplete restoration of meniscal function or irreversible cartilage degeneration may limit recovery of a favorable mechanobiological environment. Several factors have been identified as predictors of inferior outcomes following MMPRT repair, including varus alignment, bone morphology, body mass index (BMI), muscle weakness, delayed surgery, and the presence of high-grade cartilage lesions [10,11,12,13,14]. Among these, the management of concomitant focal cartilage defects remains particularly challenging. In general, MMPRTs without significant cartilage damage are considered good indications for isolated root repair, whereas extensive cartilage degeneration favors arthroplasty. In contrast, the optimal management of MMPRTs accompanied by localized high-grade cartilage defects remains controversial. Moon et al. suggested that MMPRTs accompanied by high-grade cartilage defects larger than 2.0 cm^2^ may require alternative treatment strategies beyond bone marrow stimulation procedures [14].

In cases of subchondral insufficiency fracture of the knee (SIFK), grades 1 and 2 can be managed successfully with pullout repair alone, whereas grades 3 and 4 generally require surgical interventions such as high tibial osteotomy (HTO) or unicompartmental knee arthroplasty (UKA) [15,16,17]. For MMPRT with high-grade cartilage defects, osteochondral autograft transplantation (OAT) or autologous chondrocyte implantation (ACI) may also be a potential treatment option, an alternative to bone marrow stimulation procedures [18]. OAT has been reported worldwide as another reconstructive option for cartilage defects, and its effectiveness for localized cartilage lesions is already well established [19,20]. These studies correspond to the criteria applied in the present study, with an accepted upper size limit for OAT of approximately 4.0 cm^2^. For MMPRT with varus lower limb alignment and high-grade cartilage defects, HTO or UKA may be preferred [21,22]. HTO and UKA for MMPRT have also been reported to yield favorable clinical outcomes [15,23,24]. However, compared to bone marrow stimulation procedures, HTO and UKA are more invasive and sacrifice native joint structures, which may be undesirable, particularly in younger or biologically active patients [25,26].

MMPRTs typically occur in older patients with varus-aligned knees. In rare but clinically relevant scenarios, MMPRTs occur in patients with neutral-to-mild varus or even valgus alignment who present with localized, high-grade cartilage defects that exceed the indications for bone marrow stimulation yet do not warrant osteotomy or arthroplasty. Currently, there is no clear consensus or treatment recommendation for patients with MMPRT with well-aligned lower limbs and concomitant localized high-grade cartilage defects. For this specific population, no clear consensus exists regarding the optimal joint-preserving strategy. In such cases, a combined intra-articular approach incorporating pullout repair and OAT may represent a feasible joint-preserving strategy. The combination of transtibial pullout repair and OAT represents an integrated strategy grounded in bioengineering and regenerative orthopedics. By restoring meniscal hoop tension and reducing excessive contact pressures [1,2], root repair may re-establish a biomechanical environment conducive to chondrocyte mechanotransduction and extracellular matrix homeostasis, thereby enhancing the biological potential of concomitant cartilage restoration procedures. In this context, OAT not only reconstructs focal full-thickness defects structurally but may also benefit from the normalization of joint loading achieved through root repair [1,2]. While root repair seeks to re-establish meniscal load distribution and joint biomechanics, OAT provides structural cartilage reconstruction using autologous osteochondral tissue, addressing focal full-thickness defects at a biological and material level. Thus, this approach extends beyond symptomatic treatment and reflects a tissue-preserving and regenerative concept aimed at delaying arthroplasty in carefully selected patients. Therefore, pullout repair combined with OAT may represent a joint-preserving strategy that bridges the gap between isolated root repair—with or without bone marrow stimulation—and more invasive procedures such as corrective osteotomy or arthroplasty, especially in relatively young patients seeking to preserve their native joint. However, to date, clinical data regarding this combined approach for MMPRTs are lacking.

Therefore, the purpose of this study was to describe the clinical outcomes of pullout repair combined with OAT for MMPRTs with focal cartilage defects and to compare them with those of pullout repair alone and UKA in a treatment-stratified cohort. Given the exploratory nature of this retrospective study, we hypothesized that postoperative clinical outcomes would improve compared with preoperative status in all treatment groups, while acknowledging that differences in final outcomes would be interpreted descriptively due to differences in baseline characteristics and small sample size.

## 2. Materials and Methods

### 2.1. Study Design and Patients

This study was approved by the Ethics Committee of our institution (#1857), and informed consent was obtained from all patients. The study was conducted in accordance with the principles of the Declaration of Helsinki. This was an exploratory retrospective study. We retrospectively investigated patients who underwent surgical treatment for MMPRT at our institution between April 2015 and December 2019. A total of 173 patients underwent surgical treatment for MMPRT during this period. After exclusion of patients with less than 2 years of postoperative follow-up, 150 patients were included in the final analysis. Patients were categorized into three groups according to the surgical strategy selected based on predefined clinical indications: pullout repair combined with OAT (Group O), pullout repair alone (Group P), and UKA (Group U). Treatment allocation was based on indication rather than randomization, reflecting routine clinical decision-making. Age at the time of surgery, sex, height, weight, and BMI were recorded for each patient.

### 2.2. Surgical Indications and Treatment Allocation

Surgical treatment for MMPRT was determined based on radiographic findings, limb alignment, cartilage status, and patient-specific factors. Indications for pullout repair included Kellgren–Lawrence (KL) grade ≤ 2, femorotibial angle (FTA) ≤ 180°, BMI < 30 kg/m^2^, and the presence of localized cartilage lesions. In principle, patients with International Cartilage Repair Society (ICRS) grade ≤ 2 cartilage lesions underwent pullout repair alone. Patients with high-grade focal cartilage lesions (ICRS grade ≥ 3) measuring > 1.5 cm^2^ and ≤ 4.0 cm^2^ were considered for pullout repair combined with OAT, provided that limb alignment was neutral to mild varus (FTA < 178°). These thresholds were selected based on previously published literature defining the generally treatable surface area for OAT [19,20] and lesion depths unlikely to achieve spontaneous healing after isolated pullout repair [14,18], thereby serving as practical indication criteria in the present study. Patients with cartilage lesions ≤ 1.5 cm^2^ treated with bone marrow stimulation were excluded from this study. When patients presented with high-grade cartilage lesions and unfavorable alignment (FTA ≥ 178°), conservative treatment was initially attempted. UKA was subsequently performed in cases of persistent symptoms. SIFK was identified in one knee in Group O and was not observed in the remaining cases. No patients underwent high tibial osteotomy during the study period. Given this indication-based treatment allocation, the three groups differed in baseline characteristics and disease severity.

### 2.3. Surgical Techniques and Postoperative Rehabilitation

The surgical technique was determined preoperatively based on lower limb alignment and MRI findings, and no intraoperative modifications to the planned procedure were made. Pullout repair was performed arthroscopically using either the FasT-Fix (Smith & Nephew, London, UK)-dependent modified Mason–Allen suture (F-MMA) or two simple stitches (TSS) technique [4,27]. Briefly, F-MMA or TSS was used to grasp the posterior horn and root. Tibial fixation of the sutures was performed using a double-spike plate (Meira, Nagoya, Aichi, Japan) or a bioabsorbable screw (Smith & Nephew, London, UK) at 20–30° knee flexion with an initial tension of 20–30 N.

Postoperatively, the patients were initially kept non-weight-bearing with a knee immobilizer for two weeks. Between two and four weeks, knee flexion exercises gradually increased under partial weight-bearing conditions. After five or six weeks, the patients were permitted full weight-bearing and knee flexion of 120°.

**Figure 1:**
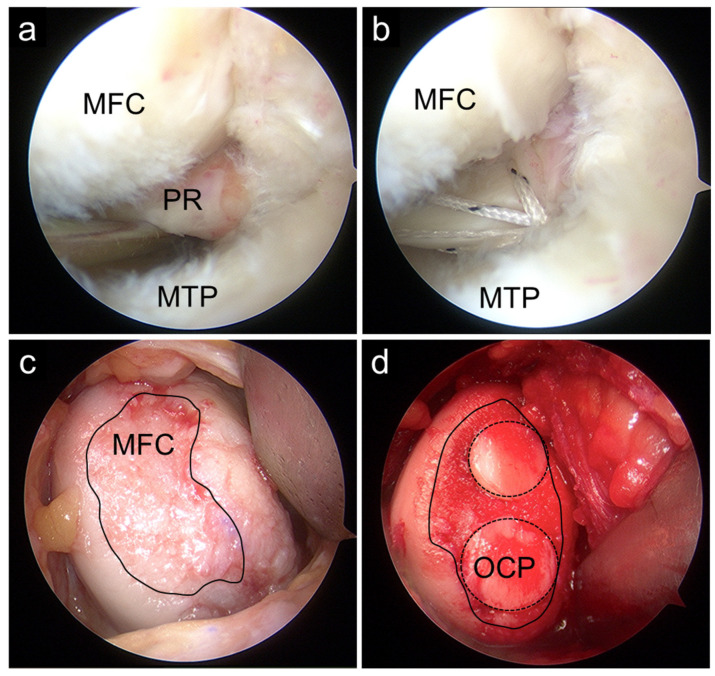
Intraoperative arthroscopic findings. Pullout repair using the two simple stitches technique (**a**,**b**) was combined with osteochondral autograft transplantation (**c**,**d**). Solid black circles indicate cartilage lesions, and dashed black circles indicate the OCP. MFC, medial femoral condyle; MTP, medial tibial plateau; OCP, osteochondral plug; PR, posterior root.

When indicated, OAT was performed via the medial parapatellar approach. Osteochondral plugs harvested from the femoral trochlea were transplanted into the cartilage defect on the medial femoral condyle (Figure 1). In combined procedures, rehabilitation was modified to protect the cartilage graft. Partial weight-bearing was initiated at 20 kg at 3 weeks postoperatively and increased incrementally by 20 kg per week. Range of motion (ROM) exercises were initiated two weeks postoperatively, starting from 0–30° and gradually advancing to 0–60° and 0–90°, and eventually to full ROM at weekly intervals.

In contrast, patients in the UKA group were allowed immediate full weight-bearing and unrestricted ROM from the first postoperative day. Because of the small sample size, matching according to differences in rehabilitation protocols was not performed.

### 2.4. Radiographic Assessments

Preoperative plain knee radiographs were used to evaluate the KL grade, FTA, medial proximal tibial angle, and medial posterior tibial slope. Magnetic resonance imaging (MRI) examinations were performed using either an Achieva 1.5 T scanner (Philips, Amsterdam, The Netherlands) or an Oasis 1.2 T open MRI scanner (Hitachi Medical, Kashiwa City, Chiba, Japan). Patients were scanned in a 10° knee-flexed position under non-weight-bearing conditions. Standard sequences of the Achieva included sagittal (repetition time [TR]/echo time [TE], 601/14) and coronal (TR/TE, 553/14) T2-weighted multi-echo with a 30° flip angle. The slice thickness was 3 mm with a 0.6 mm gap. The field of view was 18 cm with an acquisition matrix size of 512 × 358. For an Oasis scanner, Proton density-weighted images (PDWIs) in the coronal and sagittal planes were obtained. For PDWIs, the parameters were as follows: TR = 1718 ms, TE = 12 ms, flip angle = 90°, field of view = 16 cm, slice thickness = 4 mm, and acquisition matrix = 320 × 416.

**Figure 2:**
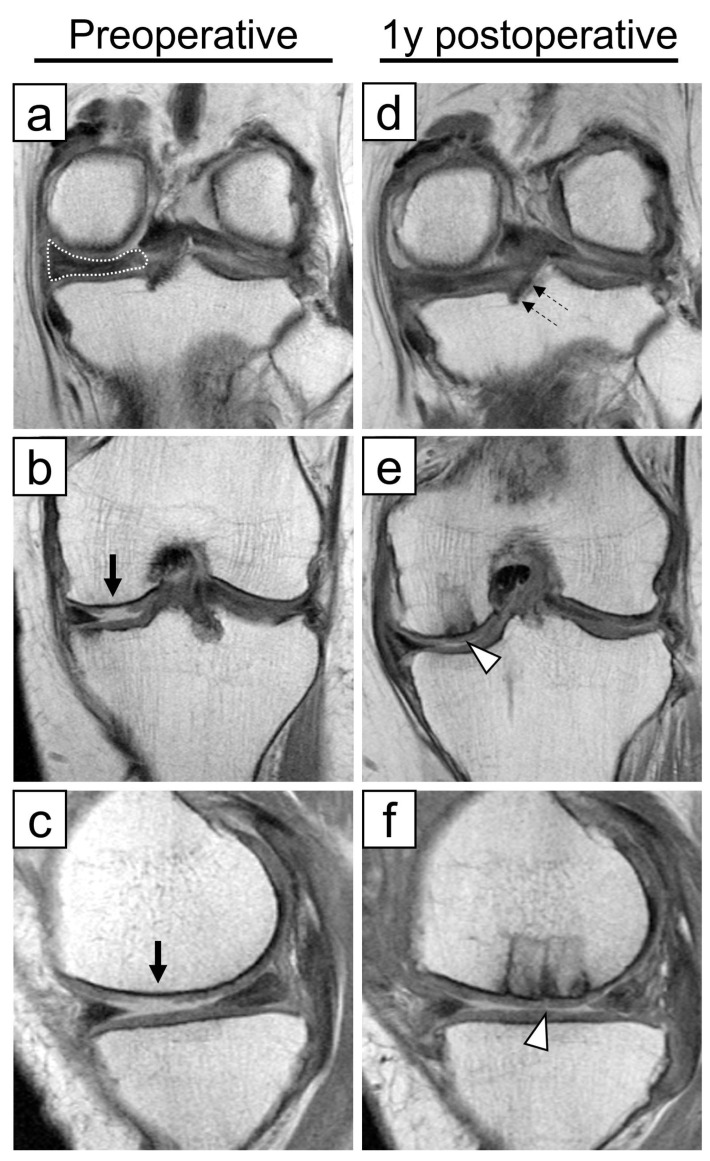
Preoperative and postoperative magnetic resonance imagings of a representative case: (**a**,**b**,**d**,**e**) coronal views; (**c**,**f**) sagittal views. A 55-year-old woman injured her left knee by twisting it on stairs. Preoperative MRI revealed a medial meniscus posterior root tear (**a**) (“giraffe neck” sign; white dotted area) and focal cartilage damage on the medial femoral condyle, approximately 2.2 cm^2^ (**b**,**c**) (black arrows). Postoperative MRI demonstrated successful repair of the posterior root, with continuity confirmed (**d**) (dashed arrow) and good incorporation of the osteochondral autograft transplantation (**e**,**f**) (white arrowhead).

MMPRT was diagnosed based on established MRI findings, including MM extrusion, cleft sign, ghost sign, and giraffe neck sign. Postoperative MRI was used to qualitatively assess continuity of the repaired posterior root and incorporation of the osteochondral graft (Figure 2). MRI assessments were descriptive and were not subjected to quantitative grading.

### 2.5. Clinical Outcome Measures

Clinical scores were assessed preoperatively (one day before surgery), 1 year postoperatively, and at the final follow-up. The primary outcome was defined as the clinical score at the final follow-up, while secondary outcomes were defined as the changes (Δ values) in each score from the preoperative baseline to the final follow-up. The following clinical outcomes were assessed: Lysholm score (0 = worst, 100 = best), Tegner activity score (0 = worst, 10 = best), International Knee Documentation Committee (IKDC) score (0 = worst, 100 = best), Visual Analogue Scale—Pain (VAS) (0 = no pain, 100 = worst possible pain), and Knee Injury and Osteoarthritis Outcome Score (KOOS). The KOOS has five sub-items: pain, symptoms, activities of daily living (ADL), quality of life, and sports and recreational function.

### 2.6. Statistical Analysis

Statistical analysis was performed using Easy R software (version 1.68) (Saitama Medical Center, Jichi Medical University, Shimotsuke city, Tochigi, Japan). Patient demographics and radiographic parameters were compared using Fisher’s exact test for sex, ANOVA for age, BMI, follow-up period, and the Kruskal–Wallis test for other continuous radiographic variables. Clinical outcomes at each time point and changes from preoperative to final follow-up were compared among the groups using the Kruskal–Wallis test. Within-group comparisons of patient-reported outcome measures (PROMs) across different time points in Groups O, P, and U were performed using the Wilcoxon signed-rank test. Statistical significance was defined as *p* < 0.05. Owing to the small sample size, no matching or adjustment for baseline differences (e.g., propensity score matching) was performed. Furthermore, given the limited sample size and the exploratory nature of the analyses, no multivariable adjustment for potential confounding factors was undertaken. Due to baseline differences among groups and the small number of patients in Group O, all analyses were considered exploratory. Outcome analyses were performed on a per-protocol basis. Patients who underwent conversion to UKA within 2 years after the index procedure (*n* = 1) were excluded from the final outcome evaluation of their original treatment group.

## 3. Results

### 3.1. Patient Characteristics

Patient demographics and radiographic characteristics are summarized in Table 1. Significant differences in sex distribution were observed among the three groups (*p* = 0.04), with a higher proportion of male patients in Group P. Preoperative FTA and KL grades also differed significantly, with Group U demonstrating greater varus alignment and more advanced radiographic OA (*p* = 0.02 and *p* < 0.01, respectively). These findings indicate that baseline disease severity, particularly in terms of radiographic OA (KL grade) and lower-limb alignment (FTA), differed substantially among the three groups at study entry. The mean follow-up duration was 5.8 years in Group O, 4.7 years in Group P, and 4.2 years in Group U, with no significant differences among the groups.

### 3.2. Clinical Outcomes over Time

**Figure 3:**
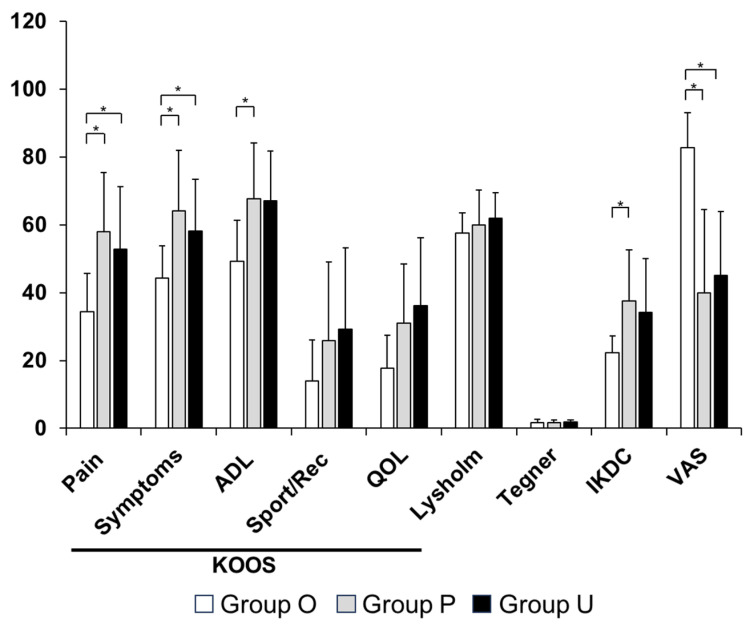
Comparison of preoperative clinical scores. Significant differences were observed among KOOS—Pain, KOOS—Symptoms, KOOS—ADL, IKDC score, and VAS. * *p* < 0.05. ADL, activities of daily living; IKDC, International Knee Documentation Committee; KOOS, Knee Injury and Osteoarthritis Outcome Score; QOL, quality of life, VAS, visual analogue scale—pain. Group O, pullout repair combined with OAT; Group P, pullout repair alone; Group U, UKA.

**Figure 4:**
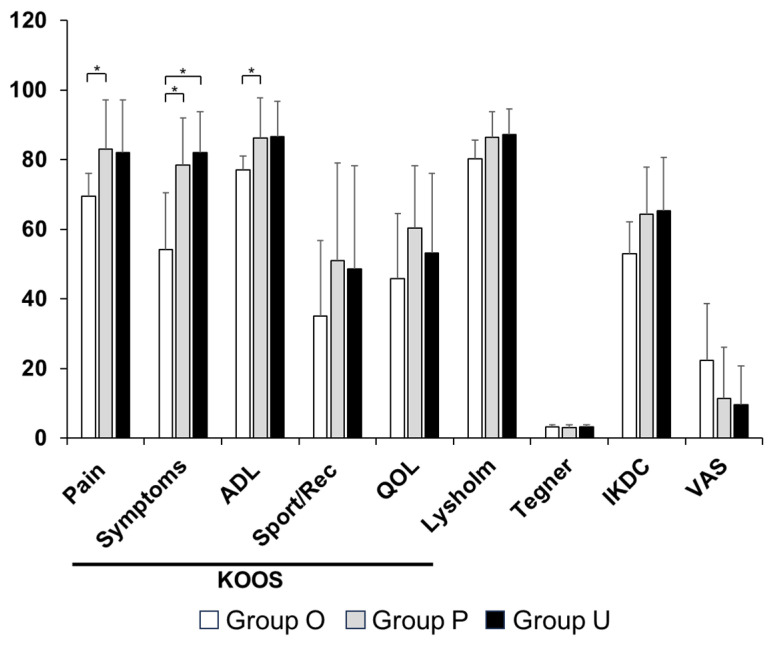
Comparison of clinical scores at 1 year postoperatively. Significant differences were observed among the KOOS—Pain, KOOS—Symptoms, and KOOS—ADL. * *p* < 0.05. ADL, activities of daily living; IKDC, International Knee Documentation Committee; KOOS, Knee Injury and Osteoarthritis Outcome Score; QOL, quality of life, VAS, visual analogue scale—pain. Group O, pullout repair combined with OAT; Group P, pullout repair alone; Group U, UKA.

**Figure 5:**
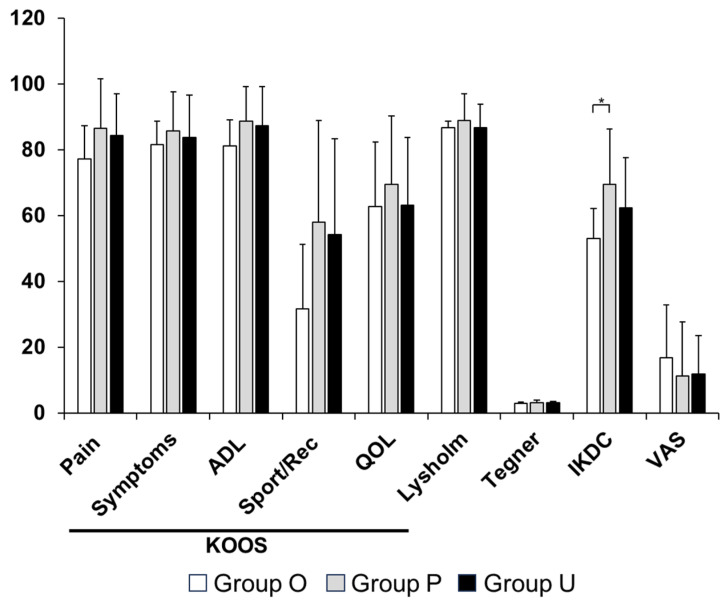
Comparison of clinical scores at the final follow-up period. Significant differences were observed for the IKDC score. * *p* < 0.01. ADL, activities of daily living; IKDC, International Knee Documentation Committee; KOOS, Knee Injury and Osteoarthritis Outcome Score; QOL, quality of life, VAS, visual analogue scale—pain. Group O, pullout repair combined with OAT; Group P, pullout repair alone; Group U, UKA.

All groups demonstrated significant improvements in clinical outcomes from the preoperative assessment to 1 year postoperatively and to final follow-up (Supplementary Tables S1–S3). At baseline, patients in Group O had significantly lower KOOS pain (*p* = 0.001), symptoms (*p* = 0.004), and ADL (*p* = 0.028) scores compared with the other groups (Figure 3). These baseline differences in patient-reported symptoms further highlight the heterogeneity in initial disease severity across the three groups. At 1 year postoperatively, Group O continued to demonstrate lower KOOS pain (*p* = 0.03), symptoms (*p* = 0.002), and ADL scores (*p* = 0.03, Figure 4). At the final follow-up, the IKDC score in Group O remained significantly lower than that in Group P (*p* = 0.007), whereas no significant differences were observed between Groups O and U (Figure 5).

### 3.3. Changes in Clinical Outcomes

**Figure 6:**
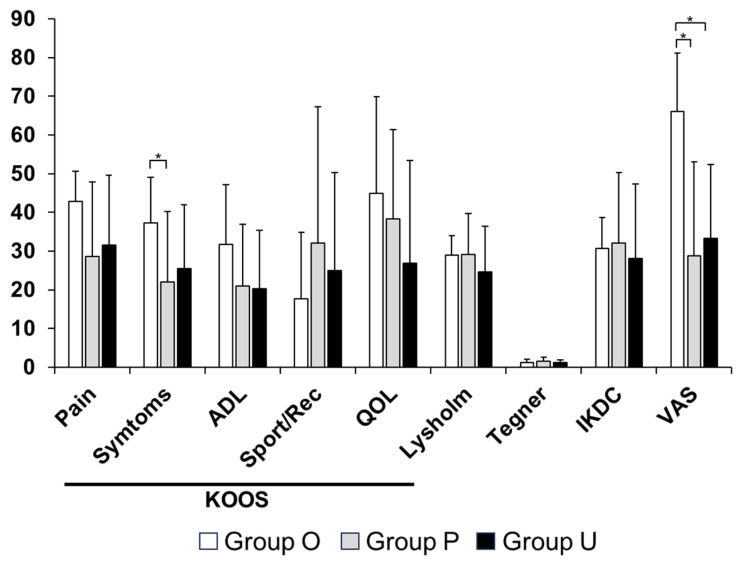
Comparison of changes in clinical scores. Significant differences were observed in the KOOS—Symptoms and VAS. * *p* < 0.05. ADL, activities of daily living; IKDC, International Knee Documentation Committee; KOOS, Knee Injury and Osteoarthritis Outcome Score; QOL, quality of life, VAS, visual analogue scale—pain. Group O, pullout repair combined with OAT; Group P, pullout repair alone; Group U, UKA.

When changes in clinical outcomes from preoperative assessment to final follow-up were analyzed, Group O demonstrated greater improvements in KOOS—Symptoms (*p* = 0.049) and VAS (*p* = 0.002) scores compared with Groups P and U (Figure 6). However, given the substantial differences in baseline radiographic severity and symptom scores among the groups, these findings should be interpreted in light of the initial heterogeneity in disease severity. Improvements in other outcome measures were observed across all groups without significant intergroup differences.

### 3.4. Radiographic and Arthroscopic Findings

**Figure 7:**
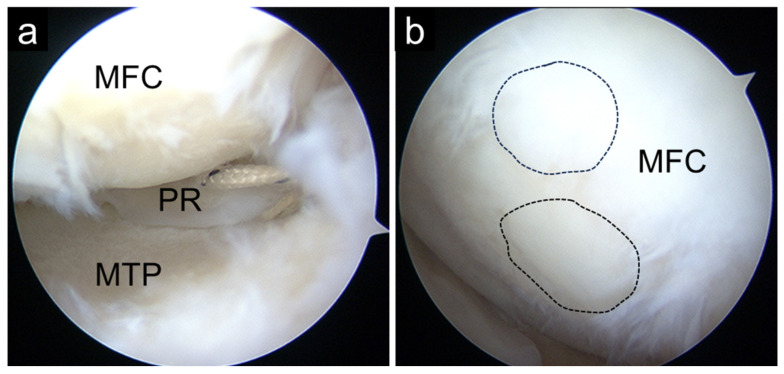
Second-look arthroscopy showing good healing of the MMPR (**a**) and successful incorporation of the osteochondral autograft transplantation plug (**b**). Dashed black circles indicate the transplanted osteochondral plug. MFC, medial femoral condyle; MM, medial meniscus; MTP, medial tibial plateau; PR, posterior root.

In Group O, the mean KL grade progressed from 1.5 preoperatively to 2.5 at final follow-up. Notably, preoperative KL grades and alignment parameters differed among the three groups, reflecting variations in baseline structural disease severity. The distribution of KL grades shifted from 0/1/2/3/4 = 0/3/3/0/0 preoperatively to 0/0/3/3/0 at final follow-up. Second-look arthroscopy performed at the time of implant removal demonstrated continuity of the repaired MM posterior root and satisfactory incorporation of the osteochondral graft (Figure 7).

### 3.5. Complications

Complications were recorded and reported descriptively without statistical comparison because of the small sample size, particularly in Group O.

In Group O (*n* = 6), no patients developed postoperative knee stiffness, poor MM healing, poor graft incorporation, graft subsidence, cartilage delamination or graft failure, or persistent donor-site pain during the postoperative period.

In Group P (*n* = 120), postoperative knee stiffness requiring arthrolysis developed in five patients. In addition, three patients demonstrated poor healing of the MM posterior root on second-look arthroscopic evaluation, defined as nonfunctional or insufficient healing. One patient experienced a traumatic twisting injury approximately one year after surgery and subsequently required conversion to UKA. This patient could not be followed for 2 years after the index procedure and was therefore excluded from the Group P outcome analysis in accordance with the per-protocol study design.

In Group U (*n* = 24), no cases of deep infection, periprosthetic fracture, implant loosening, implant subsidence, implant malposition or dislocation, symptomatic venous thromboembolism, or neurological impairment were observed. No patient developed postoperative joint stiffness requiring arthrolysis.

## 4. Discussion

The most important finding of this study was that postoperative clinical outcomes improved in all treatment groups, including patients in Group O. Although patients in Group O demonstrated inferior preoperative and early postoperative scores, the differences among groups diminished over time, and PROMs at final follow-up were largely comparable to those in Group U. Poorer baseline PROMs were observed in Groups O and U, which may have contributed to larger Δ-values in these groups, as lower baseline scores can exaggerate the magnitude of change from preoperative to follow-up assessments. The poorer baseline PROMs likely reflect the presence of concomitant cartilage lesions, indicating more advanced intra-articular pathology compared with the pullout repair-alone group. At final follow-up, IKDC scores were significantly lower in Group O compared with Group P, possibly due to the greater extent of cartilage damage in Group O [4,5,20] and the higher proportion of female patients [18,28], as both sex and cartilage status may influence postoperative PROMs. Group O demonstrated slower clinical improvement at 1 year postoperatively. This likely reflects the longer biological healing and recovery time required after OAT, as the transplanted osteochondral plugs undergo incorporation and remodeling within the joint [19,20,29,30,31,32,33]. MRI-based studies have demonstrated that plug integration is gradual, with early incorporation occurring within the first 3–6 months, but complete remodeling and structural maturation may extend up to 12 months or longer [32,33]. Therefore, the slower improvement in PROMs at 1 year in Group O is likely attributable to this biologically driven recovery process, which underscores the importance of sufficient postoperative rehabilitation and gradual load progression. Furthermore, surgical procedure selection reflected differences in patient background, including the presence and severity of cartilage lesions and variations in lower limb alignment, which likely contributed to baseline differences among groups. These findings suggest that, in carefully selected patients, a combined intra-articular joint-preserving approach may provide clinically meaningful symptom relief.

Previous studies have reported favorable outcomes following pullout repair for MMPRTs using techniques such as F-MMA and TSS [4]. Similarly, UKA has been shown to yield satisfactory outcomes for MMPRTs associated with advanced cartilage degeneration or varus alignment [4,22]. The present study extends this body of literature by providing the first clinical data, to our knowledge, on pullout repair combined with OAT performed exclusively using intra-articular procedures for MMPRTs with focal cartilage defects. The management of concomitant cartilage lesions remains a critical determinant of outcome following MMPRT repair. While bone marrow stimulation procedures are generally indicated for small defects [34], OAT [19,20] and ACI [35,36] are established options for larger or high-grade lesions. OAT and cartilage allografts are also viable options; however, allogeneic grafts may not be available in some countries, necessitating alternative treatment strategies [28].

Recent studies have demonstrated that OAT yields excellent clinical results in focal knee cartilage defects. OAT is unique in its ability to restore true hyaline cartilage, making it particularly effective in treating osteochondral lesions. Clinical trials combining OAT with adjunctive procedures, such as platelet-rich plasma scaffolding, have shown significant improvements in pain, function, and patient satisfaction, with high safety profiles and minimal complications [29]. Long-term follow-up studies have further confirmed the durability of OAT. For example, it has been reported that OAT combined with valgus high tibial osteotomy achieved 77% survivorship at nearly 19 years, with substantial improvements in Lysholm and KOOS scores and high patient satisfaction [30]. Similarly, evidence indicates that cartilage repair procedures, including OAT, help prevent the progression of knee degeneration compared with nonoperative management [31]. Although failures in cartilage restoration can occur, large registry and revision studies have highlighted that OAT remains one of the most reliable biological reconstruction techniques, provided that key factors such as alignment and meniscal integrity are addressed [37]. Overall, the literature strongly supports OAT as a safe, effective, and durable treatment option for young, active patients with symptomatic osteochondral defects. In the present study, OAT was selectively applied to patients with localized cartilage defects of limited size and controlled alignment, which likely contributed to the favorable clinical outcomes observed.

Radiographic evaluation revealed mild progression of OA in Group O over the mid-term follow-up period. This finding is consistent with previous reports indicating that MMPRT repair does not completely halt OA progression [8]. In the present cohort, the mean postoperative KL grade in Group O progressed from 1.5 to 2.5 during follow-up, suggesting that structural degeneration could not be entirely prevented despite surgical intervention. This observation aligns with prior longitudinal and biomechanical studies demonstrating that, although restoration of hoop tension and root repair can improve joint mechanics and potentially slow degenerative changes [1,2,9], it does not fully eliminate the risk of radiographic OA progression. Nevertheless, even in the presence of imaging-based OA progression, substantial improvement in pain and PROMs was achieved, indicating that symptom relief and functional recovery may allow temporary joint preservation and serve as a time-saving strategy before arthroplasty becomes necessary. In this context, joint-preserving procedures such as pullout repair combined with OAT may help bridge the therapeutic gap by improving clinical symptoms and delaying the need for joint-replacing surgery, even if complete radiographic prevention of OA progression is not attainable. Previous studies have demonstrated that preoperative KL grade is an important prognostic factor in patients with MMPRTs, with higher baseline KL grades being associated with inferior clinical outcomes and a greater likelihood of radiographic progression [10,15]. In addition, longitudinal analyses have shown that, although root repair may slow structural deterioration, progression of KL grade or medial joint space narrowing can still occur during follow-up [9]. Nevertheless, despite radiographic changes, clinical outcomes improved substantially, underscoring the well-recognized discordance between radiographic progression and PROMs. Importantly, the postoperative clinical scores in Group O were comparable to those reported in prior studies of MMPRT repair and cartilage restoration procedures performed within established indications [4,5,20,29,30].

Some limitations of this study warrant consideration. First, its retrospective design and the small number of patients in Group O limit the strength of comparative conclusions. Second, treatment allocation was based on clinical indications, resulting in inherent selection bias and baseline differences among groups. Specifically, differences in cartilage severity and lower limb alignment influenced the choice of surgical procedure and likely contributed to intergroup differences at baseline. Furthermore, because preoperative matching or statistical adjustment was not feasible due to the small sample size, differences in disease severity and patient characteristics may have influenced the observed outcomes. Therefore, the comparative results should be interpreted with caution, as residual confounding cannot be excluded. The proportion of female patients was significantly higher in the OAT group. Current literature does not provide strong or consistent evidence that sex independently influences clinical outcomes after meniscal root repair or osteochondral autograft procedures. Prior studies reporting outcomes after MMPRT repair or cartilage restoration have not identified female sex as a decisive predictor of inferior clinical outcomes [4,5,20]. Although some analyses of cartilage injury and repair have discussed demographic influences such as age and activity level [18,28], sex-specific differences remain inconclusive. Thus, while it is unlikely that female sex per se would substantially alter the biological or mechanical response to pullout repair or OAT, the observed sex imbalance in the OATS group may have contributed to differences in baseline clinical scores in this small cohort. This imbalance, in combination with other baseline differences, should be considered when interpreting the results, and larger studies are needed to further clarify the potential influence of sex in this context. Third, the follow-up period was mid-term, and longer-term data are necessary to assess durability and conversion to arthroplasty. Fourth, direct comparisons with osteotomy-based strategies were not possible. Favorable clinical outcomes have been reported in 11 knees with moderate varus alignment (hip–knee–ankle angle < 4°) treated with HTO for MMRPTs one year after surgery [38]. In their study, the average postoperative KOOS–sports and quality of life scores were 69.0 and 72.7, respectively. These were slightly higher than those in our study; however, in terms of KOOS—Pain, KOOS—Symptoms, and ADL, the average scores were 82.8, 82.4, and 88.1, respectively, which were comparable to those in our study. UKA is known to be associated with a slightly higher Hospital for Special Surgery score than HTO in their meta-analysis study [39]. However, no significant differences were detected in postoperative ROM, VAS, Lysholm, Knee Society score, Western Ontario and McMaster Universities Osteoarthritis Index, Tegner, or Oxford knee scores. Considering these findings, it is likely that similar non-inferior results would be observed in comparison with HTO. Fifth, although postoperative MRI was used to assess posterior root continuity and osteochondral plug incorporation, imaging was not performed at standardized postoperative time points across all patients, precluding quantitative MRI analysis. While all patients in Group O achieved generally satisfactory root continuity and graft integration, as illustrated in Figure 2, the lack of quantitative assessment may have influenced the interpretation of structural outcomes. In addition, second-look arthroscopy was offered to consenting patients and performed in 5 patients in Group O and 117 in Group P, primarily for removal of fixation screws, while simultaneously assessing meniscal healing and performing synovectomy as indicated. However, not all patients underwent second-look arthroscopy, and qualitative assessment of meniscal continuity could not be systematically correlated with clinical outcomes. Finally, because outcome analyses were performed on a per-protocol basis, the results may underestimate the true failure rate of pullout repair in routine clinical practice. Given the rarity of MMPRTs accompanied by focal cartilage defects in neutral-to-mild varus alignment, larger multicenter studies will be required to validate these preliminary findings.

Despite these limitations, this study provides preliminary evidence that pullout repair combined with OAT is a feasible joint-preserving option for a highly selected subset of patients with MMPRTs and localized cartilage damage who may otherwise progress to arthroplasty. This approach may help bridge the treatment gap between isolated MMPRT repair and joint-replacing procedures. Further prospective studies with larger cohorts and longer follow-up are warranted.

## 5. Conclusions

Postoperative outcomes improved in all groups (O, P, and U). Pullout repair combined with OAT yielded favorable mid-term PROMs in a carefully selected group of patients with localized cartilage defects. Mild radiographic progression of OA was observed; however, structural deterioration did not preclude meaningful clinical improvement. In cases undergoing second-look arthroscopy, satisfactory graft incorporation and root continuity were generally confirmed. As arthroscopy was not performed in all patients, correlation with clinical outcomes could not be fully assessed. The combination of meniscal root repair and OAT integrates biomechanical restoration with biological cartilage repair, supporting a tissue-preserving, regenerative approach in selected patients.

As an exploratory study, the impact of indication-based treatment allocation and the resulting selection bias should be explicitly acknowledged. Although definitive comparative conclusions cannot be drawn due to baseline differences and a small sample size, this combined intra-articular approach may offer a feasible joint-preserving option for selected patients who might otherwise progress to UKA. Long-term follow-up and larger prospective studies are necessary to clarify the durability and optimal indications of this technique.

## Figures and Tables

**Table 1:** Preoperative patients’ demographic data.

Characteristic	Group O	Group P	Group U	*p*-Value
Patients	6	120	24	
Age (years)	61.7 ± 11.6	63.3 ± 8.9	68.1 ± 6.8	0.934
Sex, male/female	1/5	27/93	5/19	0.043 *
Body mass index (kg/m^2^)	26.8 ± 5.5	25.9 ± 4.2	25.2 ± 3.3	0.635
Femorotibial angle (°)	176.8 ± 1.1	177.4 ± 1.7	178.5 ± 2.0	0.016 *
Medial posterior tibial slope (°)	9.7 ± 3.2	9.7 ± 3.0	10.0 ± 3.6	0.773
Medial proximal tibial angle (°)	84.8 ± 0.9	85.0 ± 1.6	85.1 ± 1.6	0.070
Kellgren-Lawrence grade, 0/1/2/3/4	0/3/3/0/0	0/44/65/11/0	3/7/12/2	<0.001 *
Follow-up period (years)	5.8 ± 1.2	4.7 ± 1.5	4.2 ± 1.4	0.051

Values are presented as mean ± standard deviation or number. Sex was tested by Fisher’s exact test. Age, BMI, and follow-up period were tested by ANOVA. Preoperative femorotibial angle, medial posterior tibial slope, medial proximal tibial angle, and Kellgren-Lawrence grade were tested using the Kruskal–Wallis test. * *p* < 0.05.

## Data Availability

The data presented in this study are available from the corresponding author upon reasonable request, in consideration of participant privacy.

## Supplementary Materials

The following supporting information can be downloaded at: https://www.mdpi.com/article/10.3390/bioengineering13030343/s1, Table S1. Clinical scores of group O. Table S2. Clinical scores of group P. Table S3. Clinical scores of group U.

## Abbreviations

The following abbreviations are used in this manuscript:

ACI	Autologous chondrocyte implantation
ADL	Activities of daily living
BMI	Body mass index
F-MMA	FasT-Fix -dependent modified Mason-Allen suture
FTA	Femorotibial angle
HTO	High tibial osteotomy
ICRS	International Cartilage Repair Society
IKDC	International Knee Documentation Committee
KL	Kellgren–Lawrence
KOOS	Knee Injury and Osteoarthritis Outcome Score
MM	Medial meniscus
MMPRT	Medial meniscus posterior root tear
MRI	Magnetic resonance imaging
OA	Osteoarthritis
OAT	Osteochondral autograft transplantation
PDWI	Proton density-weighted images
SIFK	Subchondral insufficiency fracture of the knee
TE	Echo time
TR	Repetition time
TSS	Two simple stitches
UKA	Unicompartmental knee arthroplasty
VAS	Visual Analogue Scale
