# Supplementary material for: Mid-Term Clinical Outcomes of Pullout Repair Combined with Osteochondral Autograft Transplantation for Medial Meniscus Posterior Root Tears with Focal Cartilage Defects: A Treatment-Stratified Cohort Study
Source: Bioengineering (Basel). 2026 Mar 16;13(3):343. doi: 10.3390/bioengineering13030343 (PMC13024123; doi:10.3390/bioengineering13030343)
Supplement: Supplementary file 1 [file bioengineering-13-00343-s001.zip › supplementary_table_S1.pdf]

**Supplementary Table S1.** Clinical scores of group O

| Clinical score | Preoperative | 1 year | Final follow up | p-value Pre/1Y | p-value 1Y/Final | p-value Pre/Final |
|---|---|---|---|---|---|---|
| KOOS score | | | | | | |
| Pain | 34.3±11.3 | 69.5±6.5 | 77.1±10.1 | 0.031* | 0.178 | 0.031* |
| Symptoms | 44.3±9.4 | 54.2±16.3 | 81.6±7.0 | 0.313 | 0.036* | 0.031* |
| ADL | 49.3±12.1 | 77.2±3.8 | 81.1±8.0 | 0.035* | 0.0563 | 0.059 |
| Sports / Rec. | 14.0±12.0 | 35±21.8 | 31.7±19.5 | 0.098 | 0.399 | 0.094 |
| QOL | 17.7±9.7 | 45.8±18.7 | 62.6±19.7 | 0.031* | 0.063 | 0.031* |
| Lysholm score | 57.7±5.9 | 80.3±5.3 | 86.7±2.1 | 0.035* | 0.115 | 0.031* |
| Tegner score | 1.7±0.9 | 3.2±0.7 | 2.8±0.4 | 0.089 | 0.424 | 0.048* |
| IKDC score | 22.3±5.0 | 52.9±9.3 | 53.1±9.1 | 0.031* | 1 | 0.031* |
| VAS scale | 82.8±10.2 | 22.3±16.2 | 16.8±16.0 | 0.031* | 0.688 | 0.031* |

Values are presented as the mean ± standard deviation or number.

Abbreviations: KOOS, Knee Injury and Osteoarthritis Outcome Score; IKDC, International Knee Documentation Committee.

Every score was tested by Wilcoxon's signed rank test.

* $p < 0.05$.
