# Supplementary material for: Mid-Term Clinical Outcomes of Pullout Repair Combined with Osteochondral Autograft Transplantation for Medial Meniscus Posterior Root Tears with Focal Cartilage Defects: A Treatment-Stratified Cohort Study
Source: Bioengineering (Basel). 2026 Mar 16;13(3):343. doi: 10.3390/bioengineering13030343 (PMC13024123; doi:10.3390/bioengineering13030343)
Supplement: Supplementary file 1 [file bioengineering-13-00343-s001.zip › supplementary_table_S2.pdf]

Supplementary Table S2. Clinical scores of group P

Clinical score	Preoperative	1 year	Final follow up	p-value Pre/1Y	p-value 1Y/Final	p-value Pre/Final
KOOS score						
Pain	57.9±17.6	83.0±14.2	86.5±15.0	<0.001*	<0.001*	<0.001*
Symptoms	64.1±17.8	78.6±13.5	85.6±12.1	<0.001*	<0.001*	<0.001*
ADL	67.6±16.5	86.2±11.6	88.6±10.7	<0.001*	0.003*	<0.001*
Sports / Rec.	23.1±29.3	51.0±27.9	58.0±30.8	<0.001*	0.003*	<0.001*
QOL	31.1±17.4	60.3±18.0	69.4±20.8	<0.001*	<0.001*	<0.001*
Lysholm score	59.9±10.4	86.4±7.3	89.0±7.9	<0.001*	<0.001*	<0.001*
Tegner score	1.67±0.9	3.1±0.7	3.2±0.7	<0.001*	0.0897	<0.001*
IKDC score	37.5±15.2	64.4±13.5	69.5±16.7	<0.001*	<0.001*	<0.001*
VAS scale	40.0±24.5	11.5±14.5	11.2±16.5	<0.001*	0.104	<0.001*

Values are presented as the mean ± standard deviation or number.

Abbreviations: KOOS, Knee Injury and Osteoarthritis Outcome Score; IKDC, International Knee Documentation Committee.

Every score was tested by Wilcoxon's signed rank test.

* $p < 0.05$.
