# Supplementary material for: Mid-Term Clinical Outcomes of Pullout Repair Combined with Osteochondral Autograft Transplantation for Medial Meniscus Posterior Root Tears with Focal Cartilage Defects: A Treatment-Stratified Cohort Study
Source: Bioengineering (Basel). 2026 Mar 16;13(3):343. doi: 10.3390/bioengineering13030343 (PMC13024123; doi:10.3390/bioengineering13030343)
Supplement: Supplementary file 1 [file bioengineering-13-00343-s001.zip › supplementary_table_S3.pdf]

Supplementary Table S3. Clinical scores of group U

Clinical score	Preoperative	1 year	Final follow up	p-value Pre/1Y	p-value 1Y/Final	p-value Pre/Final
KOOS score						
Pain	52.8±18.4	82.1±15.0	84.3±12.7	<0.001*	<0.001*	<0.001*
Symptoms	58.3±15.2	82.1±11.6	83.7±12.9	<0.001*	<0.001*	<0.001*
ADL	67.1±14.7±	86.7±10.0	87.3±11.9	<0.001*	0.003*	<0.001*
Sports / Rec.	29.3±23.9	48.9±29.7	54.2±29.2	<0.001*	0.003*	<0.001*
QOL	36.1±20.1	53.2±22.9	63.0±20.6	<0.001*	<0.001*	<0.001*
Lysholm score	62.0±7.5	87.3±7.3	86.7±7.1	<0.001*	<0.001*	<0.001*
Tegner score	1.8±0.6	3.2±0.7	3.0±0.5	<0.001*	0.090	<0.001*
IKDC score	34.2±15.9	65.3±15.2	62.3±15.3	<0.001*	<0.001*	<0.001*
VAS scale	45.0±18.9	9.7±11.1	11.8±11.7	<0.001*	0.104	<0.001*

Values are presented as the mean ± standard deviation or number.

Abbreviations: KOOS, Knee Injury and Osteoarthritis Outcome Score; IKDC, International Knee Documentation Committee.

Every score was tested by Wilcoxon's signed rank test.

* $p < 0.05$.
